# Weekly screening supports terminating nosocomial transmissions of vancomycin-resistant enterococci on an oncologic ward – a retrospective analysis

**DOI:** 10.1186/s13756-017-0206-z

**Published:** 2017-05-16

**Authors:** Stefanie Kampmeier, Dennis Knaack, Annelene Kossow, Stefanie Willems, Christoph Schliemann, Wolfgang E. Berdel, Frank Kipp, Alexander Mellmann

**Affiliations:** 1 Institute of Hygiene, University Hospital Münster, Robert-Koch-Strasse 41, 48149 Münster, Germany; 2 Institute of Medical Microbiology, University Hospital Münster, Domagkstrasse 10, 48149 Münster, Germany; 3 Department of Medicine A, Haematology and Oncology, University Hospital Münster, Albert-Schweitzer-Campus 1, 48149 Münster, Germany; 4 Present address: Institute of Hygiene, DRK Kliniken Berlin, Drontheimer Str. 39–40, 13359 Berlin, Germany

**Keywords:** Vancomycin-resistant enterococci, *E. faecium*, Screening, Infection control bundle strategies, Outbreak, Whole genome sequencing

## Abstract

**Background:**

To investigate the impact of weekly screening within the bundle of infection control measures to terminate vancomycin-resistant enterococci (VRE) transmissions on an oncologic ward.

**Methods:**

A cluster of 12 VRE colonisation and five infections was detected on an oncologic ward between January and April 2015. Subsequently, the VRE point prevalence was detected and, as part of a the bundle of infection control strategies to terminate the VRE cluster, we isolated affected patients, performed hand hygiene training among staff on ward, increased observations by infection control specialists, intensified surface disinfection, used personal protective equipment and initiated an admission screening in May 2015. After a further nosocomial VRE infection in August 2015, a weekly screening strategy of all oncology patients on the respective ward was established while admission screening was continued. Whole genome sequencing (WGS)-based typing was applied to determine the clonal relationship of isolated strains.

**Results:**

Initially, 12 of 29 patients were VRE colonised; of these 10 were hospital-acquired. During May to August, on average 7 of 40 patients were detected to be VRE colonised per week during the admission screening, showing no significant decline compared to the initial situation. WGS-based typing revealed five different clusters of which three were due to *vanB*- and two *vanA*-positive enterococci. After an additional weekly screening was established, the number of colonised patients significantly declined to 1/53 and no further nosocomial cases were detected.

**Conclusions:**

Weekly screening helped to differentiate between nosocomial and community-acquired VRE cases resulting in earlier infection control strategies on epidemic situations for a successful termination of nosocomial VRE transmissions.

## Background

Vancomycin-resistant enterococci (VRE) are important causes of healthcare associated infections [1, 2]. Acquisition of VRE has been associated with prolonged hospital stay and duration of previous hospitalization, neutropenia, antibiotic treatment, exposure to high-dose corticosteroids and immunosuppression [3–6]. Patients with hematologic malignancies have many of these predisposing factors and are at high-risk for VRE colonisation and infection.

Recent studies report an increase in VRE outbreaks on wards, hosting immunocompromised patients [7–9]. Mathematical modelling estimated a basic reproductive number R_0_ of 1.32, underlining the epidemic potential of VRE [10]. Via shedding VRE, colonised patients serve as potential sources for transmission on other patients, healthcare workers or surfaces [4]. Limiting the spread of VRE requires infection control bundle strategies such as antibiotic stewardship, patient isolation, enhanced hand hygiene, surface disinfection, and increased active surveillance [11, 12].

Screening to detect VRE carriage in risk patients is usually used to identify previously unrecognized cases on a ward in order to prevent further nosocomial spread and subsequent infections of VRE. Distinguishing community-acquired VRE cases from cases transmitted in the hospital is often difficult. Appropriate screening strategies can help to differentiate among these cases and to detect clusters of VRE. International recommendations prefer active rather than passive screening methods in hospitals [13] but due to imprecise definitions, currently performed screening strategies diverge, depending on the respective hospital. In current studies, VRE screening is mainly considered within the context of preventive activities [14]. Here, we investigate the impact of weekly VRE screening within the bundle of infection control measures to terminate VRE outbreaks on an oncologic ward.

## Methods

### Outbreak detection, screening and infection control measures

In the 1500-bed University Hospital Muenster, routine surveillance, i.e. regular review of patients’ charts and microbiological test results, detected five VRE infections on the hematologic/oncologic ward between January and April 2015. In addition 12 VRE colonisations could be detected coincidentally in anal swabs or stool samples in epidemiologically linked patients. As these rates exceeded the baseline of two infections and three incidentally detected colonisations every 12 months, an outbreak investigation was initiated. Subsequently, VRE point prevalence among all patients on ward was determined and environmental samples were taken. A VRE infection control strategy (hereafter called “VRE bundle strategy”) was established including the following measures: Patients were screened upon admission and contact precautions were implemented. Patients with positive VRE testing were isolated. Isolation of more than one patient in one room was performed if patients were colonized or infected with enterococci harbouring identical *vanA/B* resistance genes. Separation of toilets, showers, and water supplies was performed and previously shared bathing rooms were closed for colonised patients from this moment on. Staff was instructed to wear personal protective equipment in case of entering a patient room, consisting of gloves, surgical masks and gowns. Surface disinfection was performed initially in every room, including washrooms, patient rooms, nurses’ room, storage rooms, and staff rest rooms once a day using Perform® (Schülke & Mayr GmbH, Norderstedt, Germany). Hand hygiene training was performed among nurses, physicians, cleaning personnel, and kitchen staff. Implementation of hygienic measures was observed by infection control staff every day. A time line was compiled, documenting every patient’s VRE status on the ward. Patients with known VRE colonisations, detected during previous hospital stays, were immediately isolated in a single patient room. De-isolation was only performed in case of three negative swab samples collected in three consecutive weeks without application of any antibiotics within this period.

After an additional VRE infection (sepsis) in August 2015, a weekly screening was added to the VRE bundle strategy in order to clearly identify hospital-acquired colonisations and infections. Transmissions were classified as nosocomial colonisations or infections if they occurred >48 h after hospitalization and the initial screening was negative or not performed.

### VRE screening, culture and PCR testing methods

VRE screening was performed obtaining rectal (5 cm *ab ano*) swabs (Transwab ® m40 compliant, mwe, Corsham, Wiltshire, UK) that were applied to blood agar (Columbia sheep blood agar, Oxoid, Wesel, Germany) and a chromogenic selective agar (VRESelect™, Biorad, Hercules, California, USA) and incubated for up to 48 h at 37 °C. Bacterial species of suspected colonies were confirmed by MALDI-TOF-MS (Bruker Corporation, Bremen, Germany) and antibiotic susceptibility testing was performed and verified using VITEK®2 system (BioMérieux, Nürtingen, Germany) in accordance with the EUCAST standards for clinical breakpoints. In case of vancomycin resistance, the GenoType Enterococcus system (Hain Lifescience, Nehren, Germany) was used to differentiate vancomycin resistance genes *vanA*, *vanB*, *vanC1* and *vanC2/C3*.

### Environmental sampling and testing methods

Two series of environmental sampling were performed: first during the initial phase after transmission detection in May 2015, second after cleaning of hand contact surfaces in June 2015. Polywipes (mwe, Corsham, Wiltshire, UK) were applied on surfaces and incubated in Tryptic Soy Broth + LT (Merck Millipore, Eppelheim, Germany) for 24 h at 37 °C. Following, 10 μL of broth were applied to blood agar and VRE selective agar and incubated for 24 h at 37 °C. Suspected colonies were subcultured on blood agar and species identification was performed with the help of MALDI-TOF-MS (Bruker Corporation). Susceptibility testing for vancomycin was performed using Etest® (Bestbion GmbH, Liofilchem, Italy) and evaluated in accordance with the EUCAST standards for clinical breakpoints.

### Whole genome sequence-based typing

To determine the clonal relationship of isolated VRE strains, the isolates were subjected to whole genome sequencing (WGS) using the Illumina MiSeq platform (Illumina Inc., San Diego, USA) as described previously [15]. After sequencing, quality-trimming and *de novo* assembly were performed, coding regions were compared in a gene-by-gene approach (core genome Multilocus SequenceTyping, cgMLST) [16] using the SeqSphere^+^ software version 2.0 beta (Ridom GmbH, Muenster, Germany). The clonal relationship was displayed in a minimum-spanning tree that was generated using the same software. For backwards compatibility with classical molecular typing, i. e. MLST, the MLST sequence types (ST) were extracted from the WGS data *in silico*.

### Statistical analysis

All data are expressed as absolute numbers or percentage, if not stated otherwise. Statistical analyses were performed using the Fisher’s exact test for categorical data. Statistical significance was declared at *p* < 0.05.

## Results

**Fig. 1:**
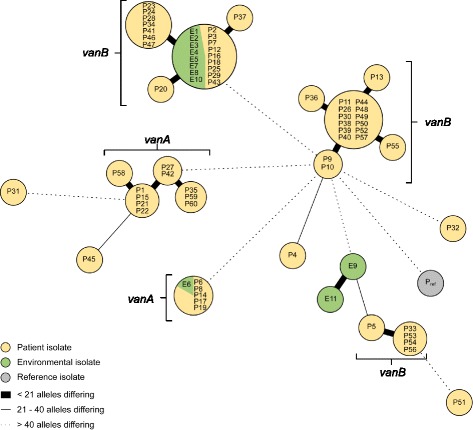
Minimum spanning tree of VRE isolates. Minimum spanning tree of 71 VRE patients (P, *yellow*) and environmental (E, *green*) isolates from haematology/oncology unit and one reference isolate (non-oncology ward, isolated in May 2015) based on 1423 cgMLST target genes, pairwise ignoring missing values. Genotypes are consecutively numbered, starting with P1 (isolated in March 2015). Each dot represents one genotype. Size of dots correlates with the number of identical genotypes. Connecting lines show the number of alleles differing between two genotypes (thick line: ≤20 alleles, thin line 21–40 alleles and dotted line >40 alleles). Whole Genome Sequencing revealed five clusters of VRE, two *vanA*-clusters and three *vanB*-clusters

Between January and April 2015 five VRE isolates from clinically relevant specimens (two blood cultures, three urines) were detected in patients on the haematology/oncology ward. Point prevalence determination of patients on the ward revealed 12 of 29 patients positive for VRE, of which 10 were *per definition* hospital-acquired, since admission screening was not performed or negative. After establishing the VRE bundle strategy, on average 7 of 40 (17.5%) patients were detected to be VRE colonised in in admission screenings, showing no significant decline compared to the initial situation. In total 30% of investigated outbreak strains harboured *vanA*, 68.3% *vanB* and 1.6% both resistance genes. MLST ST 192 (41.7%) and ST 203 (18.3%) were most prevalent (Table 1). cgMLST revealed five different VRE clusters in parallel comprising patients and environmental isolates; of these clusters three exhibited a v*anB* and two a *vanA* resistance genotype (Fig. 1).

**Table 1:** Collection dates, *van*-genotypes, MLST sequence types (all isolates) and antimicrobial resistance expression (only patient isolates) of VRE strains

Isolate no.	Collection date	*van*-genotype	MLST- ST	AMP	SAM	AX	AMC	PRL	TPZ	IPM	CIP	LEV	TEC	QD	TGC	LNZ	F	SXT	CN-HLR	S-HLR
P1	2015–03-01	vanA	203	r	r	r	r	r	r	r	r	r	r	s	s	s	r	r	+	+
P2	2015–03-18	vanB	192	r	r	r	r	r	r	r	r	r	s	s	s	s	s	r	+	−
P3	2015–04-16	vanB	192	r	r	r	r	r	r	r	r	r	s	s	s	s	s	r	+	−
P4	2015–04-26	vanA	117	r	r	r	r	r	r	r	r	r	r	s	s	s	s	r	+	−
P5	2015–05-01	vanB	80	r	r	r	r	r	r	r	r	r	s	s	s	s	s	r	−	−
P6	2015–05-22	vanA	769	r	r	r	r	r	r	r	r	r	r	s	s	s	s	r	−	+
P7	2015–05-22	vanB	192	r	r	r	r	r	r	r	r	r	s	s	s	s	s	r	+	−
P8	2015–05-23	vanA	769	r	r	r	r	r	r	r	r	r	r	s	s	s	s	r	−	+
P9	2015–05-23	vanB	117	r	r	r	r	r	r	r	r	r	s	s	s	s	r	r	+	+
P10	2015–05-23	vanA + vanB	17	r	r	r	r	r	r	r	r	r	s	s	s	s	r	r	+	+
P11	2015–05-23	vanB	N/A	r	r	r	r	r	r	r	r	r	s	s	s	s	s	r	−	+
P12	2015–05-23	vanB	192	r	r	r	r	r	r	r	r	r	s	s	s	s	s	r	−	−
P13	2015–05-24	vanB	N/A	r	r	r	r	r	r	r	r	r	s	s	s	s	s	r	−	+
P14	2015–05-25	vanA	769	r	r	r	r	r	r	r	r	r	r	s	s	s	s	r	+	+
P15	2015–05-28	vanA	203	r	r	r	r	r	r	r	r	r	r	s	s	r	r	r	+	−
P16	2015–06-02	vanB	192	r	r	r	r	r	r	r	r	r	s	s	s	s	s	r	+	−
P17	2015–06-02	vanA	769	r	r	r	r	r	r	r	r	r	r	s	s	s	s	r	+	+
P18	2015–06-02	vanB	192	r	r	r	r	r	r	r	r	r	s	s	s	s	s	r	+	−
P19	2015–06-07	vanA	769	r	r	r	r	r	r	r	r	r	r	s	s	s	s	r	+	+
P20	2015–06-08	vanB	192	r	r	r	r	r	r	r	r	r	s	s	s	s	s	r	+	−
P21	2015–06-09	vanA	203	r	r	r	r	r	r	r	r	r	r	s	r	s	r	r	−	+
P22	2015–06-09	vanA	203	r	r	r	r	r	r	r	r	r	r	s	r	s	r	r	−	+
P23	2015–06-09	vanB	192	r	r	r	r	r	r	r	r	r	s	s	s	s	s	r	−	−
P24	2015–06-09	vanB	192	r	r	r	r	r	r	r	r	r	s	s	s	s	s	r	+	−
P25	2015–06-16	vanB	192	r	r	r	r	r	r	r	r	r	s	s	s	s	s	r	+	−
P26	2015–06-16	vanB	N/A	r	r	r	r	r	r	r	r	r	s	s	s	s	s	r	−	+
P27	2015–06-17	vanA	203	r	r	r	r	r	r	r	r	r	r	s	s	s	r	r	−	+
P28	2015–06-19	vanB	192	r	r	r	r	r	r	r	r	r	s	s	s	s	s	r	+	−
P29	2015–06-23	vanB	192	r	r	r	r	r	r	r	r	r	s	s	s	s	s	r	+	−
P30	2015–06-23	vanB	N/A	r	r	r	r	r	r	r	r	r	s	s	s	s	s	r	−	+
P31	2015–07-01	vanA	192	r	r	r	r	r	r	r	r	r	r	s	s	s	r	r	+	+
P32	2015–07-02	vanB	17	r	r	r	r	r	r	r	r	r	s	s	s	s	r	r	−	+
P33	2015–07-02	vanB	80	r	r	r	r	r	r	r	r	r	s	s	s	s	s	r	+	−
P34	2015–07-07	vanB	192	r	r	r	r	r	r	r	r	r	s	s	s	s	s	r	+	−
P35	2015–07-07	vanA	203	r	r	r	r	r	r	r	r	r	r	s	s	s	r	r	+	+
P36	2015–07-12	vanB	N/A	r	r	r	r	r	r	r	r	r	s	s	s	s	s	r	−	+
P37	2015–07-16	vanB	192	r	r	r	r	r	r	r	r	r	s	s	s	s	s	r	+	−
P38	2015–07-21	vanB	N/A	r	r	r	r	r	r	r	r	r	s	s	s	s	s	r	−	+
P39	2015–07-21	vanB	N/A	r	r	r	r	r	r	r	r	r	s	s	s	s	s	r	−	+
P40	2015–07-21	vanB	N/A	r	r	r	r	r	r	r	r	r	s	s	s	s	s	r	−	+
P41	2015–07-21	vanB	192	r	r	r	r	r	r	r	r	r	s	s	s	s	s	r	+	+
P42	2015–07-21	vanA	203	r	r	r	r	r	r	r	r	r	r	s	s	s	r	r	+	+
P43	2015–07-30	vanB	N/A	r	r	r	r	r	r	r	r	r	s	s	s	s	s	r	+	−
P44	2015–07-30	vanB	N/A	r	r	r	r	r	r	r	r	r	s	s	s	s	s	r	+	+
P45	2015–07-30	vanA	203	r	r	r	r	r	r	r	r	r	r	s	s	s	r	r	−	+
P46	2015–08-06	vanB	N/A	r	r	r	r	r	r	r	r	r	s	s	s	s	s	r	+	−
P47	2015–08-06	vanB	192	r	r	r	r	r	r	r	r	r	s	s	s	s	s	r	+	−
P48	2015–08-13	vanB	N/A	r	r	r	r	r	r	r	r	r	s	s	s	s	s	r	+	+
P49	2015–08-20	vanB	N/A	r	r	r	r	r	r	r	r	r	s	s	s	s	s	r	−	+
P50	2015–08-31	vanB	N/A	r	r	r	r	r	r	r	r	r	s	s	s	s	s	r	−	+
P51	2015–09-02	vanB	80	r	r	r	r	r	r	r	r	r	s	i	s	s	s	r	+	+
P52	2015–09-07	vanB	N/A	r	r	r	r	r	r	r	r	r	s	s	s	s	s	r	+	+
P53	2015–10-08	vanB	80	r	r	r	r	r	r	r	r	r	s	s	s	s	s	r	+	+
P54	2015–10-19	vanB	80	r	r	r	r	r	r	r	r	r	s	s	s	s	s	r	+	+
P55	2015–10-19	vanB	N/A	r	r	r	r	r	r	r	r	r	s	s	s	s	s	r	−	+
P56	2015–10-26	vanB	80	r	r	r	r	r	r	r	r	r	s	s	s	s	s	r	+	−
P57	2015–11-25	vanB	N/A	r	r	r	r	r	r	r	r	r	r	s	s	s	s	r	−	+
P58	2015–11-30	vanA	203	r	r	r	r	r	r	r	r	r	r	s	s	s	r	r	−	−
P59	2015–12-21	vanA	203	r	r	r	r	r	r	r	r	r	r	s	s	s	r	r	+	+
P60	2015–12-21	vanA	203	r	r	r	r	r	r	r	r	r	r	s	s	s	r	r	+	+
P_ref_	2015–05-15	vanA	192	r	r	r	r	r	r	r	r	r	r	s	s	s	r	r	+	+
E1	2015–05-11	vanB	192																	
E2	2015–05-11	vanB	192																	
E3	2015–05-11	vanB	192																	
E4	2015–05-11	vanB	192																	
E5	2015–05-11	vanB	769																	
E6	2015–05-11	vanA	192																	
E7	2015–05-19	vanB	192																	
E8	2015–06-02	vanB	80																	
E9	2015–06-02	vanB	192																	
E10	2015–06-02	vanB	80																	
E11	2015–06-02	vanB	N/A																	

*AMP* ampicillin, *SAM* ampicillin/sulbactam, *AX* amoxicillin, *AMC* amoxicillin/clavulanic acid, *PRL* piperacillin, *TPZ* piperacillin/tazobactam, *IPM* imipenem, *CIP* ciprofloxacin, *LEV* levofloxacin, *CN-HLR* gentamicin-high level resistance, *S-HLR* streptomycin-high level resistance, *TEC* teicoplanin, *QD* quinopristin/dalfopristin, *TGC* tigecyclin, *LNZ* linezolid, *F* nitrofurantoin, *SXT* timetoprim/sulfamethoxazole, *MLST* Multi Locus Sequence Typing, *VRE* Vancomycin resistant enterococci

*r* resistant, *s* susceptible

**Fig. 2:**
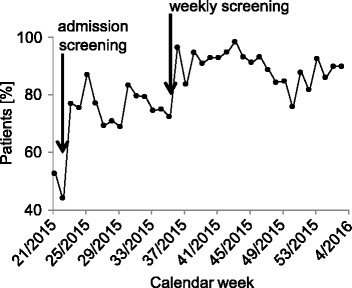
VRE screening during May 2015 – January 2016. Average percentage of screened patients in whom screening was indicated between May 2015 and January 2016 per calendar week. *Arrows* indicate the starting points of the admission screening in May and weekly screening in the end of August 2015

**Fig. 3:**
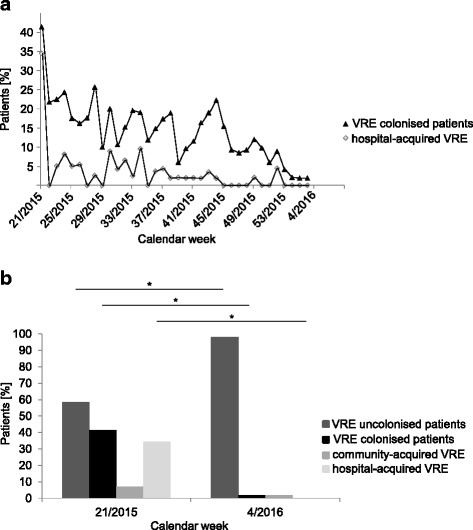
Colonised, community- and hospital-acquired VRE patients between May 2015 and January 2016. **a** Average percentage of colonised and hospital- acquired VRE patients between May 2015 and January 2016 per calendar week. **b** Statistical comparison of uncolonised patients, colonised patients, community-acquired and hospital-acquired VRE patients before (May 2015) and at the end (January 2016) of performing VRE bundle strategies plus additional weekly screening

To evaluate effectiveness of weekly screening in addition to the VRE bundle strategy, percentages of screened, colonised, hospital-acquired and isolated patients were analysed: After implementation of admission screening in May 2015 the percentage of screened patients was 76%. With establishing weekly screening in the end of August 2015 on average 91% of all patients on ward were screened (see also Fig. 2). The number of colonised patients in January 2016 declined to 1 of 53 (~2%, *p* = 0.00001) and no further nosocomial cases were detected (*p* = 0.00001) (Fig. 3). Closely connected to this situation, the number of weekly isolated patients due to a positive VRE status declined significantly from 21 of 55 patients in May 2015 to 6 of 59 patients in January 2016 (*p* = 0.00007) (Fig. 3a). While the number of community-acquired VRE did not change remarkably comparing May 2015 to January 2016, the number of total VRE colonisations decreased significantly due to a decline in hospital-acquired VRE colonisations (Fig. 3).

## Discussion

Within the here presented study, spread of VRE on the oncologic ward was suspected, in particular after the initial point prevalence of VRE colonisations was determined 41.3%. During the following months (May–August) VRE colonisation rate on admission was 17.5%, which clearly exceeds admission prevalence published elsewhere [12]. This might be due to the fact, that a high number of patients, including VRE colonised patients, was repeatedly admitted in two- or three-week- intervals for chemotherapeutic treatment. Data of detected MLST ST and *van*-genotypes, both comparable to published investigations on clinical *E. faecium* isolates [17], provided a first hint, that different VRE clones were circulating on this ward. Of note, we found MLST ST 192 and ST 203, which are the most causative STs of German VRE outbreaks [17–19], to be most prevalent on this ward. Interestingly, MLST ST 117 or ST 80, as e.g. found during VRE outbreaks in German neighbouring countries (Denmark, the Netherlands) [16], did not play a major role in our setting. cgMLST, which can be used to precisely monitor transmission rates [15], helped to illustrate, that spread of different VRE clones had taken place.. This spread was ended after establishment of weekly screening in addition to common infection control bundle strategies. Published studies mostly evaluate screening within non-outbreak settings. Here, active screening on high-risk wards was shown to reduce the incidence of VRE bacteraemia and colonisations compared to wards also hosting high-risk patients but not performing active screening [20]. The results of the present study indicate that even in situations of VRE spread, weekly screening supports reduction of VRE colonisations and infections. The combination of VRE bundle strategies plus weekly screening turned out to be most effective. These findings surprise, as weekly screening *per se* does not reduce transmission rates [21]. Screening approaches alone can help uncover undetected VRE colonisations and identify potential patient reservoirs early in order to prevent transmission of VRE within a bundle of infection control strategies, while the application of infection control measures as hand hygiene and contact precautions has more significant effect on terminating VRE transmissions [13, 22]. A possible explanation why weekly screening has a direct influence in terminating spread of VRE might be that personnel’s awareness is increased, if patients are weekly monitored and detected. In addition, since VRE cannot only be transmitted via direct or indirect contact but can also be selected due to the use of antibiotic agents, weekly screening serves the possibility of a colonisation follow up. Standard and extended hygienic measures can than concentrated and expanded when appropriate and VRE colonisation dynamics among patients on ward is more apparent.

Our study has limitations. First, we did not perform a case-control study. Hence, other interventions on the ward could have been responsible for decreased VRE colonisations. However, since all other measures of our VRE bundle were already implemented when transmission began and were not changed during the intervention period, this fact has a presumably small influence on the presented results. Second, we used different methods for susceptibility testing of either clinical or environmental samples. Routinely clinical samples are tested via VITEK 2 in order to have other therapeutic possibilities for patient’s treatment. In contrast, environmental samples, that are collected to clarify the distribution of VRE on surfaces, are tested for vancomycin resistance via Etest® since no therapeutic interventions are derived from these findings. Nevertheless, susceptibility evaluation was done in accordance with the latest EUCAST criteria, thereby creating comparable results. Third, we investigated the clonal spread of VRE only using cgMLST, therefore, we could not exclude whether *vanA* or *vanB* were passed via horizontal gene transfer from one strain to another as investigated elsewhere [18, 23].

## Conclusion

Our study support the hypothesis, that active screening reduces the incidence of VRE infections and colonisations in high-risk patients by the early uncovering of nosocomial transmissions prior their appearance in clinical samples. Therefore, a weekly screening should be considered as part of a bundle strategy for the successful termination of VRE outbreak situations.

## Abbreviations

cgMLST: Core genome multilocus sequence typing
VRE: Vancomycin-resistant enterococci
WGS: Whole genome sequencing
